# Integrated Redox Proteomic Analysis Highlights New Mechanisms of Sensitivity to Silver Nanoparticles

**DOI:** 10.1016/j.mcpro.2021.100073

**Published:** 2021-03-20

**Authors:** Reetta Holmila, Hanzhi Wu, Jingyun Lee, Allen W. Tsang, Ravi Singh, Cristina M. Furdui

**Affiliations:** 1 Section on Molecular Medicine, Department of Internal Medicine, Wake Forest School of Medicine, Winston-Salem, North Carolina, USA; 2 Wake Forest Baptist Comprehensive Cancer Center, Wake Forest Baptist Medical Center, Winston-Salem, North Carolina, USA; 3 Center for Redox Biology and Medicine, Wake Forest School of Medicine, Winston-Salem, North Carolina, USA; 4 Department of Cancer Biology, Wake Forest School of Medicine, Winston-Salem, North Carolina, USA

**Keywords:** silver nanoparticles, lung, proteomics, reversible protein oxidation, mitochondria, AgNP, silver nanoparticle, DPBS, Dulbeccos’s phosphate-buffered saline, FBS, fetal bovine serum, IPA, Ingenuity Pathway Analysis, ROS, reactive oxygen species, TEM, transmission electron microscopy, TFA, trifluoroacetic acid

## Abstract

Silver nanoparticles (AgNPs) are widely used nanomaterials in both commercial and clinical biomedical applications, but the molecular mechanisms underlying their activity remain elusive. In this study we profiled proteomics and redox proteomics changes induced by AgNPs in two lung cancer cell lines: AgNPs-sensitive Calu-1 and AgNPs-resistant NCI-H358. We show that AgNPs induce changes in protein abundance and reversible oxidation in a time and cell-line-dependent manner impacting critical cellular processes such as protein translation and modification, lipid metabolism, bioenergetics, and mitochondrial dynamics. Supporting confocal microscopy and transmission electron microscopy (TEM) data further emphasize mitochondria as a target of AgNPs toxicity differentially impacting mitochondrial networks and morphology in Calu-1 and NCI-H358 lung cells. Proteomics data are available *via* ProteomeXchange with identifier PXD021493.

## Highlights

• AgNP-sensitive cells experience broader changes in protein abundance.
• Redox proteomics reveals increased reversible oxidation in AgNP-sensitive cells.
• AgNPs alter protein translation, lipid metabolism, and bioenergetics.
• Mitochondria is identified as key target underlying AgNP toxicity.

## Graphical Abstract

**Figure:**
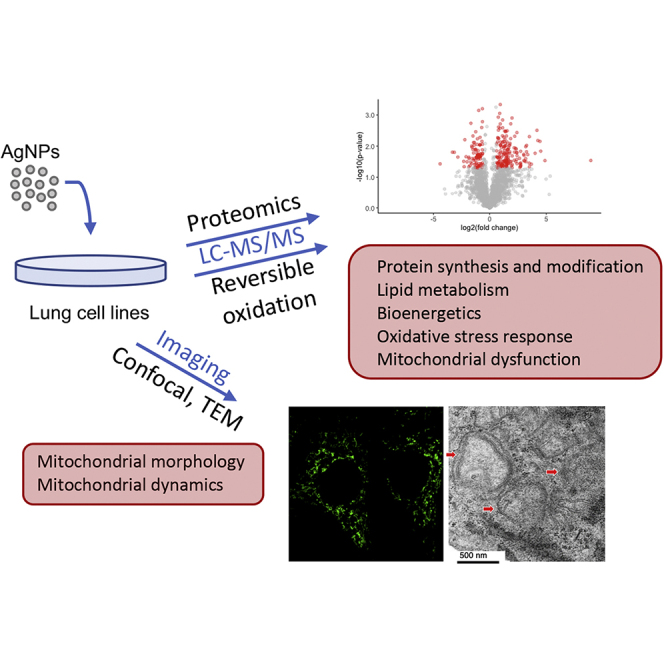

## In Brief

Silver nanoparticles (AgNPs) are widely used nanomaterials, but the molecular mechanisms underlying their activity remain elusive. Here, we show using time course studies that AgNP-sensitive lung cells experience broader and more pronounced changes in protein abundance and protein oxidation impacting protein translation and modification, lipid metabolism, bioenergetics, and mitochondrial dynamics. The findings are further validated by imaging of mitochondria using confocal microscopy and transmission electron microscopy.

Silver nanoparticles (AgNPs) are one of the most commonly used metal nanoparticles. Due to their small size, physical, chemical, optical, conductive, and antibacterial properties, they have been used to manufacture a wide variety of products for industrial, medical, and consumer applications such as implants, catheters, and wound dressings, making their release into biological systems possible (1, 2). With an overall increase in the production of AgNPs and their usage, there is the risk of an increase in exposure to humans. Indeed, there is already information about the adverse effects of high dosage, bulk, and chronic silver exposure to human health (2). Previous studies have also demonstrated that AgNPs can cause cellular toxicity, oxidative stress, autophagy, and apoptosis and decrease cellular proliferation (3, 4, 5, 6, 7, 8, 9), prompting additional investigations on their potential application as cancer therapeutics.

Mitochondria are a vital source of intracellular reactive oxygen species (ROS) regulating cellular metabolism, bioenergetics, homeostasis, and overall cell viability and function. The mitochondria actively undergo highly regulated dynamics that balance the opposing processes of fission and fusion. Fission plays a critical role in mitochondrial proliferation during cellular division, removal of damaged mitochondria *via* mitophagy, and regulation of apoptosis during cellular stress. Fusion on the other hand facilitates increased ATP production *via* oxidative phosphorylation. Dysregulation of the rates of fission and fusion can lead to mitochondrial fragmentation or elongation with broad implications on cells’ ability to meet increasing demands for energy during a time of cellular stress (10, 11, 12).

Oxidative stress has been long thought to play an essential role in the toxicity of AgNPs impacting mitochondrial membrane potential and mitochondrial redox state (5, 13, 14, 15, 16, 17, 18, 19, 20). We previously showed an increase in mitochondrial ROS and mitochondrial protein oxidation in human lung cell lines correlating with the sensitivity to AgNPs (5). However, there is considerable heterogeneity in the sensitivity of different cell lines to AgNP exposure, and it is unknown which factors drive increased sensitivity to AgNPs (3, 5, 7). Here, we aimed to identify the proteins whose expression or redox state changes upon exposure to AgNPs and quantify these using label-free relative quantitative proteomics and redox proteomics analysis in two lung cell lines, Calu-1 and NCI-H358. Based on our earlier studies (5), the Calu-1 is AgNPs-sensitive, whereas the NCI-H358 is relatively more resistant to AgNPs, and comparison of these two cell lines could help elucidate the cellular processes underlying sensitivity to AgNPs. Also two different time points, 4 h and 24 h, were used to study early and late AgNP response, similarly selected based on our previous studies (5). We demonstrate that AgNPs trigger time and cell-line-dependent changes in both protein levels and reversible protein oxidation regulating pathways involved in the cellular growth and maintenance, lipid metabolism, and mitochondrial dynamics. To our knowledge, this is the first study profiling AgNPs-induced redox changes at proteome level in human lung cells.

## Experimental Procedures

### Experimental Design and Statistical Rationale

Global proteomics and redox proteomics experiments were performed using AgNPs-sensitive Calu-1 and AgNPs-resistant NCI-H358 lung cell lines exposed to a single dose of AgNPs (20 μg/ml) for 4 h and 24 h. Additional lung cell lines, A549 and BEAS-2B, were included in confocal imaging validation studies. Label-free relative quantification was based on three biological replicates to ensure rigor and reproducibility of findings. The top significant proteins regulated by AgNPs and the representative molecular pathways were identified by applying complementary statistical methods and filtering the data by significance and fold change as detailed below. Analyses were conducted in R to produce heatmaps and bar- and volcanographs (21). Molecular pathways were identified using Ingenuity Pathway Analysis software (IPA, QIAGEN Inc).

### Cell Culture and Treatments

All four cell lines were obtained from American Type Culture Collection. Cells were cultured at 37 °C under 5% CO_2_, in RPMI-1640 medium (Gibco/Thermo Fisher Scientific) for Calu-1 and NCI-H358, in Ham’s F-12 K (Kaighn’s) medium (Gibco/Thermo Fisher Scientific) for A549 and in DMEM medium (Gibco/Thermo Fisher Scientific) for BEAS-2B. Culture medium was supplemented with 10% fetal bovine serum (FBS, Sigma-Aldrich), 100 U/ml penicillin, and 100 μg/ml streptomycin (Gibco/Thermo Fisher Scientific).

The cells were treated with a single type of 25 nm spherical AgNPs coated with polyvinylpyrrolidone (PVP). The AgNPs were obtained from nanoComposix and characterized in an earlier study (3). AgNPs were dispersed at a concentration of 5 mg/ml in Dulbeccos’s phosphate-buffered saline (DPBS) (Thermo Fisher Scientific) by bath sonication and diluted to a final concentration of 20 μg/ml in appropriate culture media.

### Proteomics and Redox Proteomics Analysis

#### Cell Culture and Protein Extraction

The cells were cultured to 90% confluence in 100 mm cell culture dishes and treated with 20 μg/ml AgNPs for 4 and 24 h as described above. After removal of media and 3× washes with cold PBS, the cells were lyzed with 500 μl of modified RIPA (mRIPA; 50 mM Tris HCl, 150 mM NaCl, 1 mM EDTA, 1% NP-40, 0.25% sodium deoxycholate, 1 mM NaF) supplemented with 10 mM MSTP ((4-(5-methanesulfonyl-[1,2,3,4]tetrazol-1-yl)-phenol); Xoder Technologies) to selectively alkylate the reduced proteins and small-molecule thiols. In this procedure, MSTP replaced the most commonly used iodoacetamide to increase compatibility with redox proteomics analysis. Iodoacetamide, N-ethylmaleimide, and other commonly used thiol alkylators react with protein sulfenic acids (-SOH at Cys residues) interfering with the quantification of this reversible oxidative modification (22). MSTP was identified as a selective alkylator of reduced protein thiols (-SH at Cys residues) meeting the needs of redox proteomics workflows (23). The lysate was incubated on ice for 30 min and centrifuged at 18,000*g* for 10 min and at 4 °C. Protein concentration was measured with the bicinchoninic acid assay and two aliquots were removed for proteomic (50 μg) and redox proteomic (100 μg) analysis. In both cases, the samples were first precipitated with cold acetone (4× volume) overnight at −20 °C and processed as described below. The treatment and protein extraction were repeated three times independently.

#### In-Solution Digestion for Proteomic Analysis

After acetone precipitation, samples were centrifuged at 16,000*g* for 5 min. The supernatant was discarded and the protein pellet was dried at room temperature for 5 min. The proteins were resuspended in solution by adding 200 μl of 50 mM NH_4_HCO_3_, followed by proteolysis with 2.5 μg trypsin (trypsin:protein = 1:20; Pierce Trypsin Protease, MS Grade, Thermo Scientific) at 37 °C overnight. The reaction was quenched with 1% formic acid (FA) and the peptides were dried using SpeedVac. Dried samples were stored at −80 °C prior to LC-MS/MS analysis.

#### Redox Proteomics—Reduction of Reversible Oxidized Thiols and Enrichment

The procedure followed published methods (24). The pellet collected by spinning the acetone precipitated samples at 16,000*g* for 5 min at 4 °C was resuspended in 150 μl resuspension buffer (50 mM HEPES, 1 mM EDTA, 0.1% SDS, pH 7.5). The samples were then reduced with 10 mM DTT at 37 °C and mixing for 1 h at 850 rpm. Excess DTT was removed using Bio-Gel P6 spin columns and the proteins were loaded on thiopropyl Sepharose resin (GE healthcare, Cat.No. 17042001), which was freshly made and preconditioned with the binding buffer (50 mM HEPES and 1 mM EDTA, pH 7.5). The tubes were then rotated at 4 °C overnight. Next day, the resin was washed with each of the following solutions: 8 M urea; 2 M NaCl; 0.1% SDS in PBS; 80% (vol/vol) acetonitrile (ACN) and 0.1% (vol/vol) trifluoroacetic acid (TFA) in H_2_O; and 50 mM NH_4_HCO_3_.

#### Redox Proteomics—On-Resin Tryptic Digestion and Elution of Enriched Cysteine-Containing Peptides

The proteins covalently bound to the thiopropyl Sepharose resin were digested at 37 °C overnight by adding 200 μl 50 mM NH_4_HCO_3_ to each tube and trypsin for a trypsin-to-protein ratio of 1:20. After digestion, the resin was again washed with the 8 M urea, 2 M NaCl, 80% ACN and 0.1% TFA, and 50 mM NH_4_HCO_3_ solutions to remove unbound peptides. Covalently bound cysteine-containing peptides were then eluted twice with 100 μl elution buffer (25 mM NH_4_HCO_3_ with 20 mM DTT) after incubation for 30 min at room temperature and mixing at 850 rpm. The resin was finally washed with 100 μl of 80% ACN/0.1% TFA to elute the remaining peptides. The peptide fractions were then combined, dried using a SpeedVac, and then stored at −80 °C for LC-MS/MS analysis.

#### LC-MS/MS Analysis

The same LC-MS/MS instrument and method were used for analysis of both proteomics and redox proteomics samples. The analysis was performed using a nanoLC (Dionex Ultimate 3000) coupled with an LTQ Orbitrap Velos Pro mass spectrometer (Thermo Scientific). The peptides were separated using a flow rate of 300 nl/min and a 2-h linear gradient consisting of mobile phase A: 95% H_2_O, 5% ACN, and 0.1% FA; and, mobile phase B: 20% H_2_O, 80% ACN, and 0.1% FA. The MS spray voltage was 1.9 kV, and the temperature of the heated capillary was 300 °C. The analysis was performed using data-dependent analysis (top-10) by alternating full-scan high-resolution MS with MS/MS analysis of fragment ions induced by collision-induced dissociation (CID) with dynamic exclusion of 30 s. Data were searched with the following parameters to identify the proteins, compile the data, and MS1 quantitation: Sequest HT algorithm within the Proteome Discoverer v2.2 (Thermo Scientific) in combination with the UniProt protein FASTA database (*Homo sapiens*, 20,258 annotated entries, Feb 2018); FT-trap instrument, parent mass error tolerance of 10 ppm; fragment mass error tolerance of 0.6 Da (monoisotopic); maximum missed cleavage of two sites using the digestion enzyme trypsin, which cleaves specifically at the C-terminal side of Lys and Arg amino acid residues; variable modifications of 15.995 Da (oxidation) on methionine, and 160.039 Da (MSTP) on cysteine. The identified peptides were validated using a reversed database search with target decoy PSM validator node under the parameters of 0.05 for maximum delta Cn, 0.01 and 0.05 for each strict and relaxed target FDR. For relative protein quantitation, the abundance was assessed by label-free quantification workflow where total peak areas of identified peptides were measured, which were normalized to the total ion current.

#### Data Analysis

For each condition, a mean value of peak area across the three replicates was calculated, and then for each time point, the AgNPs-treated conditions were compared with the untreated controls by calculating log2(fold change) and statistically tested using Student’s paired *t*-test and Pearson correlation coefficient. Analyses were conducted in R (21). Heatmaps were produced with gplots packages (25) and the bar- and volcanographs using the ggplot2 package (26). Pathway analysis was done with IPA software (QIAGEN Inc).

### Confocal Microscopy

The mitochondria were stained with 100 nM MitoTracker Green (Thermo Fisher Scientific) for 30 min at 37 °C. The cells were washed three times with DPBS (Thermo Fisher Scientific) and imaged live using a Zeiss 880 confocal microscope with Airyscan mode (laser excitation 488 nm). Fiji ImageJ (27) was used for image processing. The experiment was repeated independently three times. The brightness was adjusted and threshold was automatically defined using Otsu-method. The close-up sections were also filtered through unsharp mask and median filters to reduce blurriness with same settings for all the images. The mitochondrial networks were quantified using MiNA ImageJ plug-in (28).

### Transmission Electron Microscopy

Transmission Electron Microscopy: Calu-1 or NCI-H358 cells were plated in 6-well tissue culture dishes (10^6^ cells/well) and allowed to adhere overnight. Cells were treated with AgNPs (20 μg/ml) for 4 h. Cells were washed thoroughly in PBS and fixed in 2.5% glutaraldehyde at 4 °C overnight. Next, cells were scraped from the wells, pelleted, embedded in resin, cut into ultrathin sections (80 nm), placed on copper-coated formvar grids, and stained with osmium tetroxide by the Wake Forest Comprehensive Cancer Center Cellular Imaging Shared Resource. Cells were imaged using a Tecnai Spirit transmission electron microscope (FEI) at 80 kV.

## Results

### Global Analysis of Proteomic and Redox Proteomic Data

**Fig. 1:**
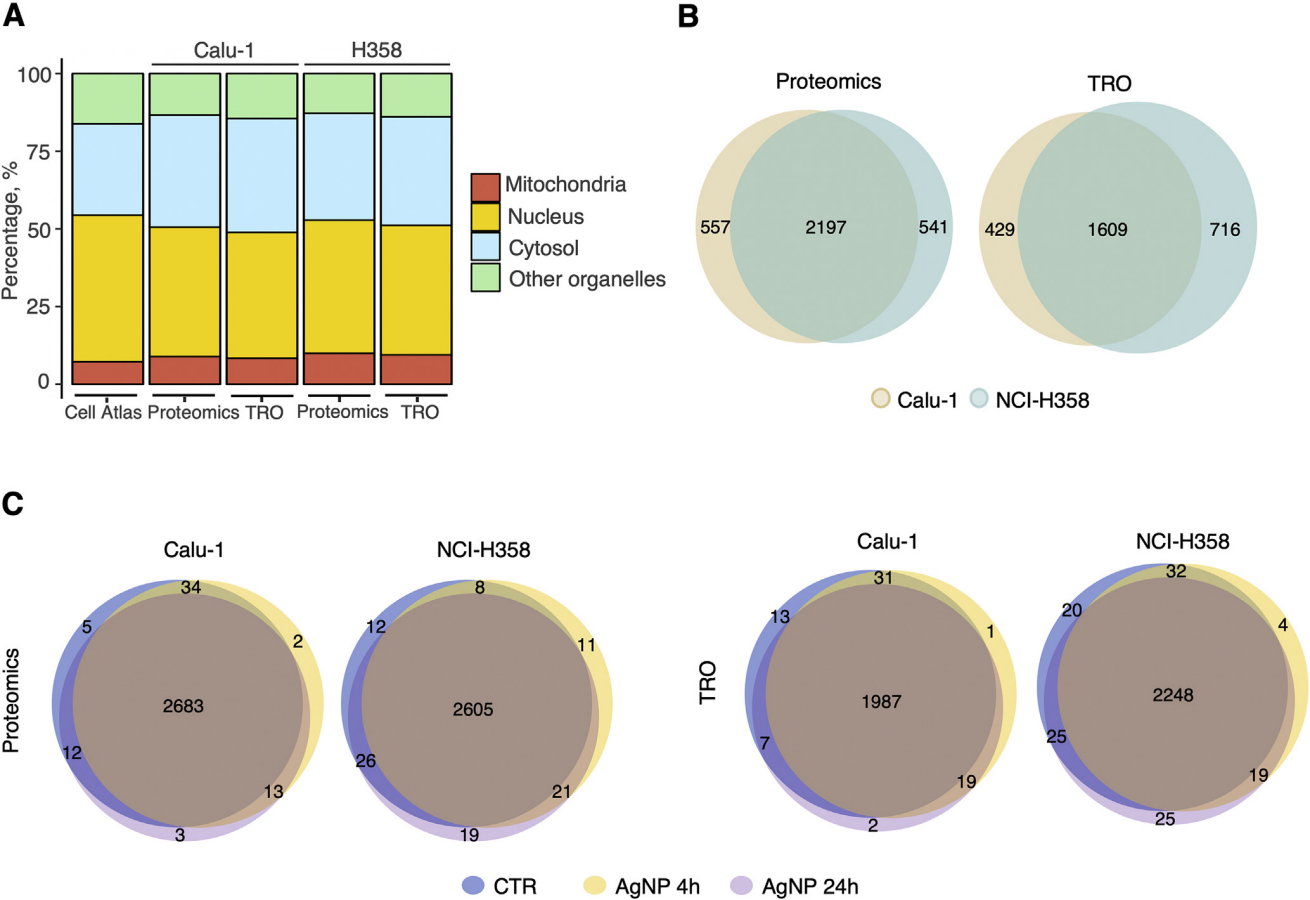
**Overview of the proteomic and redox proteomic data.***A*, distribution of protein subcellular localization compared with Cell Atlas data extracted from the Human Protein Atlas. *B*, Venn diagrams showing the overlap of proteins identified in the two cell lines at baseline by proteomic and redox proteomic analysis monitoring total reversible oxidation (TRO). *C*, Venn diagrams showing the overlap of proteins identified in control and AgNPs-treated cells in each cell line for both the proteomic and redox proteomic analysis (TRO).

We used global proteomic and total reversible oxidation (TRO) analysis to profile the changes in protein abundance and reversible oxidation in two lung cell lines, Calu-1 and NCI-H358 with and without AgNP treatment (20 μg/ml) for 4 and 24 h (supplemental Data S1, *A* and *B*). Approximately 2600 proteins were detected with proteomics analysis and ∼2000 proteins using TRO analysis. Overall, the data showed a distribution of protein locations consistent with the Human Protein Atlas (29) (Human Protein Atlas available from http://www.proteinatlas.org) (Fig. 1*A*) and with overlapping coverage for both Calu-1 and NCI-H358 cells (Fig. 1*B*). There was also a nearly complete overlap of proteins detected in vehicle control and AgNPs-treated cells (Fig. 1*C*), allowing for robust statistical comparisons of cell lines and treatment conditions.

**Fig. 2:**
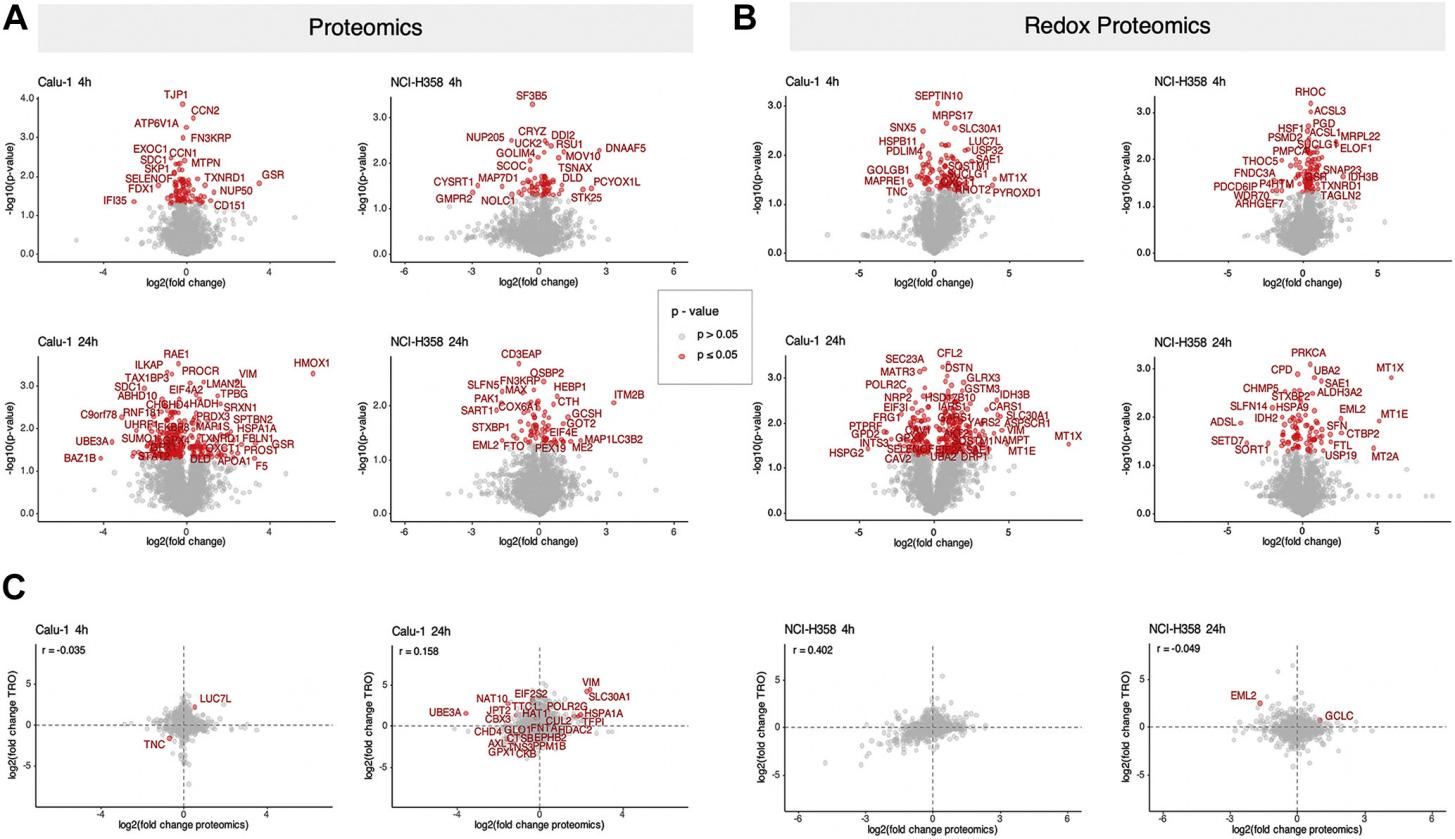
**Comparison analysis of significantly altered proteins induced by treatment with AgNPs**. *A*, volcano plots showing proteins with significantly altered abundance at 4 h and 24 h treatment with AgNPs. *B*, volcano plots showing proteins with significantly altered redox state at 4 h and 24 h treatment with AgNPs. *C*, Pearson’s correlation analysis of changes in protein abundance and protein oxidation in Calu-1 and NCI-H358 cells at 4 h and 24 h treatment with AgNPs. Data Information: *Red symbols* represent statistically significant changes (Student’s *t*-test, *p* < 0.05, n = 3) in protein abundance (*A*), redox state (*B*), and both abundance and redox state (*C*) in the two cell lines at the two treatment time points relative to their respective controls. In panel *C*, Pearson’s *r* values are listed in each graph.

Both the proteomic and the redox proteomic data showed more proteins with altered abundance and redox state in the AgNP sensitive Calu-1 cells compared with the NCI-H358 cells particularly at 24 h treatment (Fig. 2; supplemental Data S2) (*p* < 0.05). At the 4 h time point, 96 proteins were significantly altered at the protein level and 110 proteins at the redox level in Calu-1 cells, compared with 74 and 100 proteins, respectively for the NCI-H358 cells. At the 24 h time point, a higher number of proteins were significantly changed by treatment in Calu-1 (297 and 307 proteins in the proteomics and TRO analysis, respectively), while these remain approximately the same in NCI-H358 cells (76 and 94 proteins quantified based on the proteomics and TRO analysis, respectively). In general, AgNPs induced global suppression of protein levels in Calu-1 cells, whereas in NCI-H358 global protein levels were less altered, and the distribution of suppressed and increased proteins was more balanced (Fig. 2*A*). This profile was even more accentuated in Calu-1 cells at 24 h showing significantly more proteins regulated by AgNPs. Similarly, the redox proteomic analysis showed induction of protein oxidation after 4 h of AgNP treatment in both Calu-1 and the NCI-H358 cells, whereas after 24 h more significant changes in protein oxidation were detected in Calu-1 cells (Fig. 2*B*). The same trend was noted if the data were further filtered by fold change of effects (FC > 1.5) (supplemental Fig. S1 and supplemental Data S3). We then asked if the changes in protein oxidation after 4 h and 24 h treatment were due to changes in protein abundance (Fig. 2*C*). Across data considering all proteins regardless of whether they showed statistically significant changes in abundance or oxidation with 4 or 24 h AgNPs treatment, there was no strong correlation between protein abundance and oxidation using Pearson’s correlation analysis. There were, however, exceptions and the proteins with significant changes in both oxidation and abundance are highlighted in Figure 2*C* in red. Most of these occurred in Calu-1 at 24 h treatment, the data pointing to proteins such as vimentin (VIM) showing increased abundance and oxidation (potentially linked to cellular senescence (30)), ubiquitin protein ligase 3A (UBE3A), the level of which decreased with increased oxidation consistent with previous studies reporting increased aggregation and precipitation of this protein under oxidative stress conditions (31), and the antioxidant protein glutathione peroxidase 1, GPX1, representing a subset of proteins with complex mechanisms of redox regulation (32), and which in this case is downregulated by AgNP-induced oxidation.

**Fig. 3:**
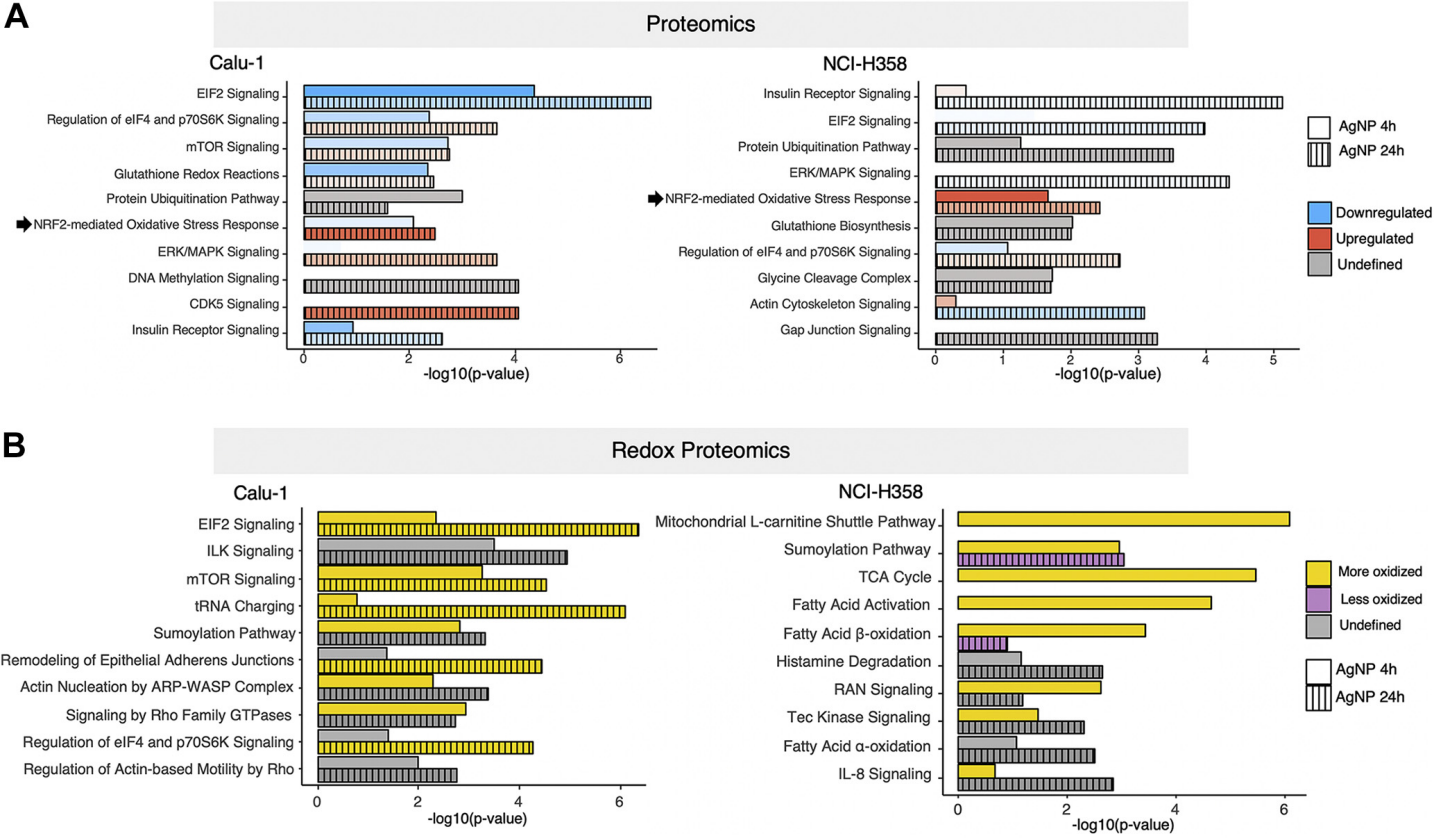
**Comparison analysis of significantly altered proteins and networks induced by treatment with AgNPs**. *A*, representative signaling and metabolic pathways identified by Ingenuity Pathway Analysis (IPA) using the subset of proteins undergoing statistically significant changes with AgNPs treatment (*p* < 0.05). *B*, representative signaling and metabolic pathways identified by IPA using the subset of proteins undergoing statistically significant changes (*p* < 0.05) in redox state with AgNPs treatment. More oxidized is defined as having most of the detected proteins oxidized and less oxidized is defined as having most of the detected proteins showing less oxidation.

The proteins with significantly changed abundance or redox state (*p* < 0.05) were imported next into the IPA to identify signaling and metabolic pathways regulated by AgNPs. Based on the proteomic data, the most affected pathways were associated with protein synthesis and posttranslational modifications, free radical scavenging, and cellular morphology, maintenance, growth, and proliferation (Fig. 3*A*). A key finding emerging from this analysis was the differential activation profile of Nrf2-mediated Oxidative Stress Response, which was strongly upregulated in NCI-H358 cells with 4 h exposure to AgNPs and remained active at the 24 h time point, while in Calu-1 cells this pathway was initially downregulated and then slightly activated over the 24 h treatment with AgNPs. Indeed, among the statistically significantly changed proteins, there were many proteins participating in antioxidant and stress-response systems such as heme oxygenase I (HMOX1), peroxiredoxin 3 (PRDX3), heat shock proteins HSPA1B and HSPA9, serine/threonine kinase 25 (STK25), metallothioneins MT1X, MT1E, and MT2A, pyridine nucleotide-disulfide oxidoreductase domain 1 (PYROXD1), GPX1, thioredoxin reductase 1 (TXNRD1), and proteins NDRG1 and selenoprotein F (SELENOF). Other proteins include various aminoacyl tRNA synthetases (supplemental Data S4 and supplemental Fig. S2), proteins associated with genomic stability and gene transcription, protein stability, endoplasmic reticulum (ER) signaling, cell cycle, and autophagy and mitophagy (functional annotation of all statistically significant proteins based on the UniProt database (33) is included in supplemental Data S2). These findings point to broad disruption of protein synthesis machinery, which is oxidatively damaged and kinetically incompetent to keep up with the demand to replenish the oxidized and/or misfolded proteins in AgNPs-treated Calu-1 cells (34).

The most significant finding emerging from IPA analysis of redox proteomic data was also the regulation of predominantly metabolic pathways in NCI-H358 cells, while signaling and protein homeostasis pathways dominate the Calu-1 redox profile (Fig. 3*B*). This observation is consistent with studies over the last years demonstrating a survival advantage of cells that are most able to quickly adapt metabolism to respond to environmental stresses including oxidative stress (35).

### AgNPs Regulation of Mitochondrial Proteins

**Fig. 4:**
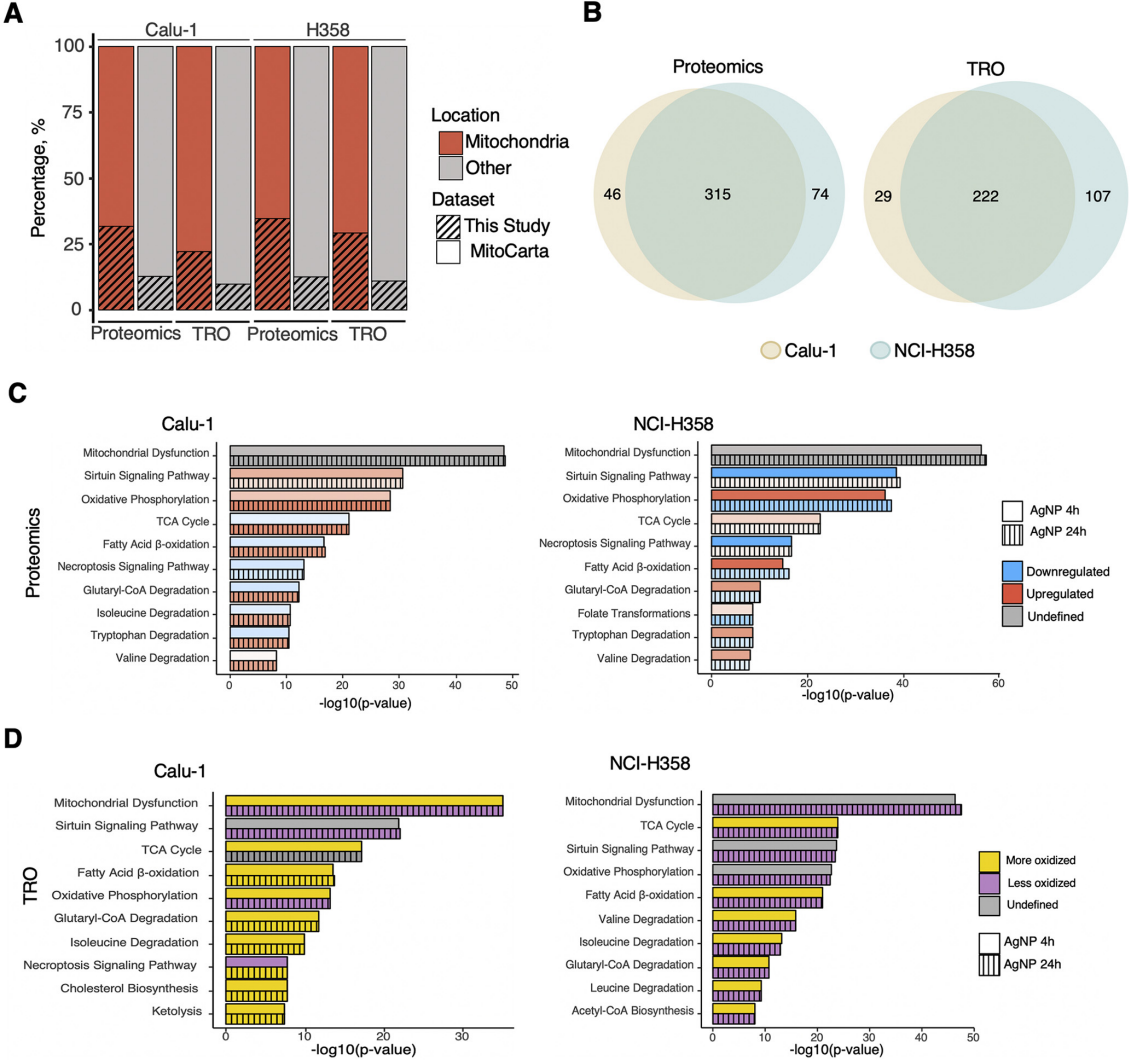
**AgNPs regulation of mitochondrial pathways**. *A*, overlap of proteomics and TRO datasets with the MitoCarta. The *bars* represent the subset of mitochondrial (*red*) and non-mitochondrial (*gray*) proteins in the MitoCarta, and the *dashed pattern* represents the percent overlap with our study using the full data set. *B*, Venn diagrams showing the overlap between the mitochondrial proteins identified in Calu-1 and NCI-H358 cells extracted from proteomic and redox proteomic (TRO) analysis. *C*, representative signaling and metabolic pathways identified by Ingenuity Pathway Analysis (IPA) of mitochondrial proteins. *D*, representative signaling and metabolic pathways identified by focusing the IPA on the redox-regulated mitochondrial proteins. More oxidized is defined as having most of the detected proteins oxidized and less oxidized is defined as having most of the detected proteins showing less oxidation.

In an earlier study, we showed that exposure to AgNPs increases mitochondrial protein sulfenylation in AgNP-sensitive cells negatively impacting mitochondrial respiration (5). Also, the proteomic and redox proteomic analysis above pointed to regulation of mitochondrial proteins as being significantly correlated with exposure and sensitivity to AgNPs. Therefore, we further focused our data analysis specifically on mitochondrial proteins (supplemental Data S5). Overall, our data sets consisted of about 32% of the mitochondrial proteins listed in the MitoCarta (36) for the proteomic data and 22% for the redox proteomic data (Fig. 4*A*), with >60% overlap between the NCI-H358 and Calu-1 cells (Fig. 4*B*). Processing of these data with IPA revealed mitochondrial dysfunction as a top pathway emerging from both data sets (Fig. 4, *C* and *D*), followed by mechanistically related sirtuin signaling and energy and lipid metabolism pathways. In general, at the basal level mitochondrial proteins were expressed at a higher level in the NCI-H358 cells relative to the Calu-1 cells (supplemental Fig. S3) and AgNPs induced statistically significant changes in the expression and redox state of many mitochondrial proteins in both cell lines. Noteworthy, there was a clear difference in the temporal profiles of energy metabolism pathways (sirtuin, OXPHOS, TCA cycle, fatty acid β-oxidation) regulated by AgNPs in Calu-1 and NCI-H358 cells (supplemental Fig. S4). For example, sirtuin signaling was upregulated in Calu-1 at 4 h of exposure to AgNPs, while it was downregulated in NCI-H358. On the other hand, at the same time point fatty acids β-oxidation was upregulated in NCI-H358 and downregulated in Calu-1 followed by reverse trends at 24 h. Considering the differential effects of SIRT3 and SIRT4 mitochondrial sirtuins in the regulation of fatty acids β-oxidation with SIRT3 functioning as activator and SIRT4 downregulating this pathway (37, 38), the results suggest SIRT4 activity as the dominant regulator of fatty acids β-oxidation in both cell lines with short exposure times, replaced by SIRT3 activity at more prolonged exposure to AgNPs. This emerging hypothesis remains to be validated in future experimental studies.

### Mitochondrial Dynamics

**Fig. 5:**
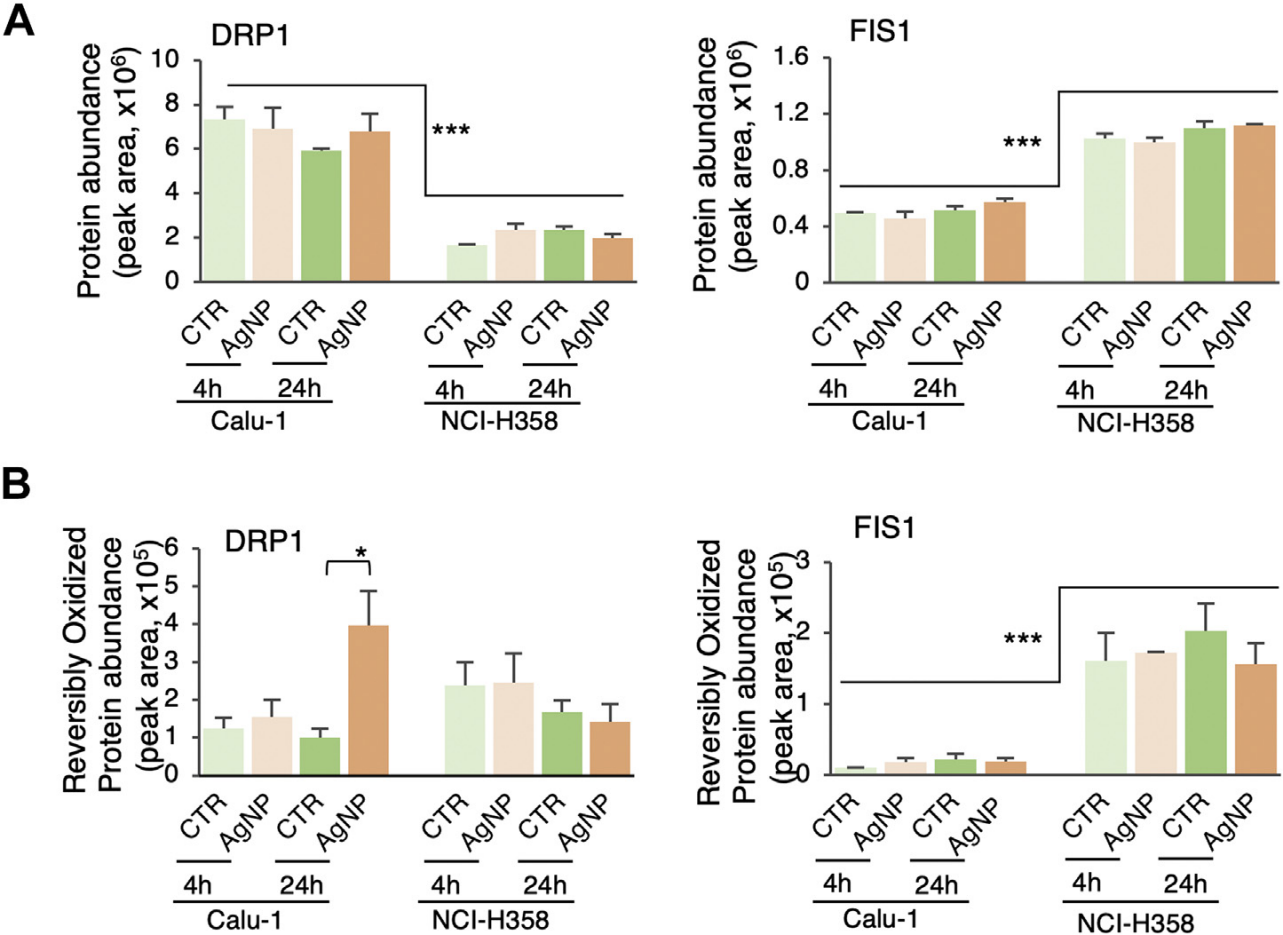
**DRP1 and FIS1 protein abundance and redox profiles across experimental conditions**. *A*, protein abundance profiles of mitochondrial fission proteins DRP1 and FIS1 extracted from the proteomic data. *B*, reversible oxidation profiles of mitochondrial fission proteins DRP1 and FIS1 extracted from the redox proteomic (TRO) data. Data Information: In both panels (*A* and *B*), data are presented as mean ± SEM. ∗∗∗*p* ≤ 0.001, ∗*p* 0.01 to 0.05 (Student's *t*-test; N = 3 for all protein abundance data with the exception of FIS1 where N = 2 for 24 h control; N = 2 for all data extracted from redox proteomic analysis with the exception of Calu-1 4 h control (N = 1), Calu-1 4 h AgNPs (N = 3), NCI-H358 4 h control (N = 3), and NCI-H358 24 h AgNPs (N = 3)). The lower N is due to missing data in the redox proteomic analysis and removal of outliers.

Mitochondrial dynamics play an essential role in mitochondrial quality control, and it is regulated by proteins involved in mitochondrial biogenesis, fusion, and fission processes. While we did not detect mitochondrial fusion proteins in either the proteomic or redox proteomic data sets, mitochondrial fission proteins DRP1 and FIS1 were detected in both data sets across baseline and treatment conditions (Fig. 5, *A* and *B*). There were statistically significant differences in these proteins between the cell lines and their response to AgNPs. At the basal level and across treatment conditions (Fig. 5*A*), the DRP1 and FIS1 showed opposite protein abundance profiles with DRP1 being higher in Calu-1 compared with NCI-H358 cells, and FIS1 being lower in Calu-1 relative to NCI-H358 cells (3- to 4-fold, *p* < 0.001). Treatment with AgNPs did not induce a statistically significant change in protein abundance for either protein regardless of cell line or treatment time. Redox proteomic analysis showed changes in the redox state of DRP1 with AgNPs treatment in Calu-1 but not NCI-H358 cells (Fig. 5*B*). In Calu-1 cells, there was a slight increase in DRP1 oxidation with 4 h treatment and a much more significant increase at 24 h treatment. In contrast, DRP1 oxidation did not change significantly in NCI-H358 at either of the two time points relative to the respective controls. Treatment with AgNPs did not affect the FIS1 redox state with treatment in either of the cell lines, but there was overall higher FIS1 oxidation in NCI-H358 (∼10-fold, *p* < 0.001).

**Fig. 6:**
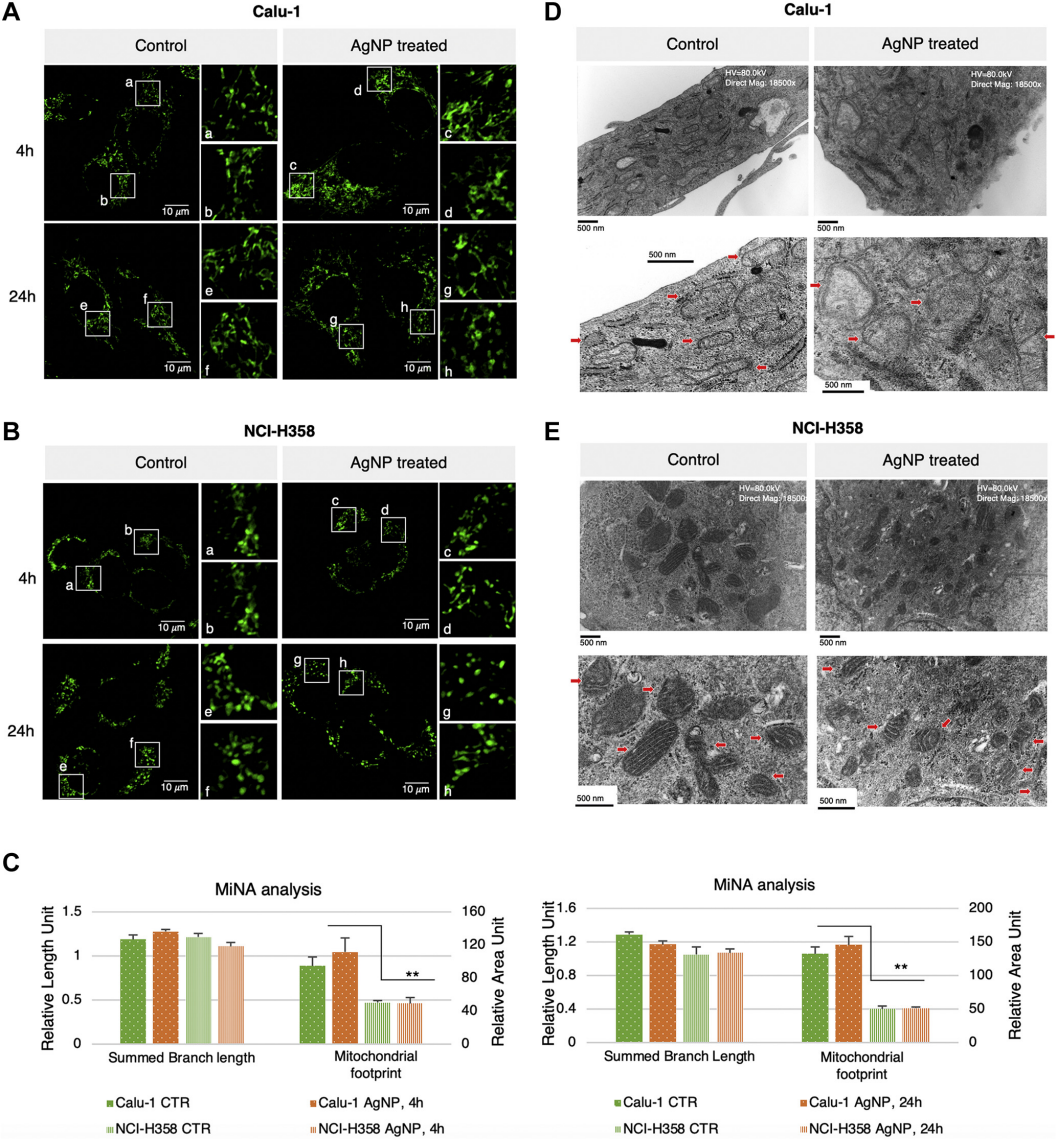
**Confocal and TEM imaging of mitochondrial networks**. *A* and *B*, MitoTracker Green staining of Calu-1 (*A*) and NCI-H358 (*B*) controls and AgNPs treated cells collected at 63× magnification. The *white squares* show the areas for the AiryScan zoomed-in sections. *C*, quantification of mitochondrial footprint using MiNA ImageJ add-on. The mitochondrial footprint represents area of the image consumed by mitochondrial signal and summed branch length is mean of the sum of the lengths of branches for each independent structure. Data are presented as mean ± SEM. ∗∗*p* 0.01 to 0.05 (Student's *t*-test; N = 3 for 4 h treatment, and N = 2 for 24 h treatment). *D* and *E*, representative TEM images of Calu-1 (*D*) and NCI-H358 (*E*) mitochondria in control and 4 h AgNPs treatment. *Red arrows* point to key mitochondrial substructures.

Given the contradictory proteomics profiles of DRP1 and FIS1 and the lack of fusion proteins in the proteomics data, next we employed imaging to determine the mitochondrial phenotype of Calu-1 *versus* NCI-H358 at baseline and with AgNPs treatment. Mitochondrial networks were first visualized by staining cells (control and cells treated for 4 h and 24 h with AgNPs) with MitoTracker Green followed by confocal imaging (Fig. 6). After 4 h exposure to AgNPs, the Calu-1 mitochondria networks showed more connectivity and elongation, which then progressed to fractured mitochondrial networks at 24 h when there was also significant cell death (Fig. 6*A*). When the networks were analyzed using MiNA ImageJ add-on (28), there was an increase in the mitochondrial footprint at both timepoints after treatment with AgNPs, signaling widening of the mitochondrial network, but these changes were not statistically significant (Fig. 6*C*). For untreated NCI-H358 cells the mitochondrial footprint was significantly smaller than the untreated Calu-1 cells (*p* 0.043), and the mitochondrial network remained unchanged upon treatment with AgNPs (Fig. 6, *B* and *C*). Further analysis using TEM imaging indicates that mitochondria in untreated Calu-1 cells (Fig. 6*D*) are more elongated, larger, and have a less dense matrix and more poorly defined cristae compared with mitochondria in untreated NCI-H358 cells (Fig. 6*E*). These findings explain and are consistent with the larger mitochondrial footprint of Calu-1 cells detected by confocal imaging. The results are also well aligned with our previous reports of increased mitochondrial mass and spare respiratory capacity of Calu-1 compared with NCI-H358 cells (5). Following exposure to AgNPs, increased mitochondrial swelling and loss of cristae were apparent in Calu-1 cells, but no changes were observable in NCI-H358 cells (Fig. 6, *D* and *E*, respectively). The swelling along with expression and redox profiles of FIS1 and DRP1 indicate a process where Calu-1 cells are attempting to repair the oxidative damage induced by AgNPs through fission and perhaps mitophagy. However, the rupture of the mitochondrial outer membrane noted in the TEM images after 4 h of treatment with AgNPs suggests a predominant path that leads instead to apoptosis. The disruption of the mitochondrial outer membrane structural integrity is consistent with decreased coupling efficiency for ATP production in Calu-1 cells compared with NCI-H358 cells both at baseline and with AgNPs treatment observed in our earlier studies (5).

## Discussion

Lung is the probable target of environmental or occupational AgNPs exposure through inhalation. Previous studies in lung cell lines have shown that AgNPs induce changes in the levels of ROS, mitochondrial protein oxidation, decrease in cell proliferation, and increase in cell death (3, 4, 5, 6, 7, 8, 9). However, what was also evident from these reported studies is that cells vary greatly in their response to AgNPs with some displaying resistance to treatment. The mechanisms underlying the differential sensitivity to AgNPs remain elusive, and their elucidation is critical both to advance their application as cancer therapeutics and to alleviate the toxicity of environmental AgNPs.

Even though the increase in oxidative stress and mitochondrial damage induced by exposure to AgNPs has been well documented in various experimental settings (1), time course analysis of proteome-wide oxidative changes in human cells has not been reported to our knowledge. Thus, in this study we performed side-by-side proteomic and redox proteomic analyses at short (4 h) and long (24 h) exposure to AgNPs to identify molecular features associated with sensitivity to AgNPs in two human lung cancer cell lines displaying differential sensitivity to AgNPs, Calu-1 and NCI-H358. These cell lines were selected based on our earlier studies (5) showing increased tolerance to AgNPs treatment in NCI-H358 relative to Calu-1 cells.

Previous reports focusing on the mechanisms of AgNPs exposure across a variety of healthy and cancer cell lines have identified regulation of fatty acid metabolism (39, 40, 41), mitochondrial electron transport chain (41, 42), ubiquitin-proteasome pathway (40, 42, 43, 44), TCA cycle (40, 44), integrin signaling (44), cytokine signaling (44), EIF2 signaling (43, 45), NRF2 signaling (43, 45), glycolysis (43), gluconeogenesis (43), and sirtuin signaling (45). Our proteomics data aligned well with these findings showing alterations in many of these pathways, but also pointed to differential regulation of these pathways in the AgNPs-sensitive and -resistant cells. For example, EIF2 signaling regulating both global and specific mRNA translation and involved in cellular proteotoxic stress response was downregulated in Calu-1 cells after 4 h and 24 h of AgNPs exposure, but it was upregulated in NCI-H358 cells at 4 h treatment. Similarly, upregulation of sirtuin signaling and downregulation of fatty acids β-oxidation in Calu-1 upon short-term exposure to AgNPs followed by reverse regulation with long-term exposure (downregulation of sirtuin signaling and upregulation of fatty acids β-oxidation) and complementary profile in NCI-H358 suggest an intricate temporal interplay of SIRT4 and SIRT3 activities as presented briefly in the Results. We propose a potential scenario by which SIRT4 acts as dominant regulator of fatty acids β-oxidation at short times (4 h) being replaced potentially by SIRT3 activity at longer exposure to AgNPs. In Calu-1, SIRT4 upregulation with 4 h exposure to AgNPs would lead to decreased fatty acids β-oxidation and utilization of glycolysis as primary fuel for mitochondrial ATP generation. This scenario is consistent with higher mitochondrial oxidative state (ROS and protein oxidation) in Calu-1 relative to NCI-H358 cells (5), but this complex regulatory mechanism remains to be investigated in future studies.

There have not been many reports of redox regulation by AgNPs at the proteome level, except a decrease in the total content of reduced thiols identified in myelin isolated from AgNP-exposed rats (46). Previously, using confocal imaging analysis we found a significant increase in mitochondrial protein sulfenylation in Calu-1 cells treated with AgNPs for 4 h resulting in a substantial decrease in cell proliferation at 24 h exposure, whereas mitochondrial protein sulfenylation was virtually unaffected in NCI-H358 cells under these conditions (5). The formation of protein sulfenic acids when thiols react with H_2_O_2_ is the first step in many oxidative processes and detection of protein sulfenylation implies the presence of oxidative modifications (47). Here, we used LC-MS/MS to identify the proteins undergoing reversible oxidation with AgNPs treatment in Calu-1 and NCI-H358 cells. For both cell lines there was an increase in protein oxidation at 4 h treatment with AgNPs, but whereas in the AgNP-sensitive Calu-1 cells, the number of proteins with altered oxidation state increased with longer 24 h exposure, in the NCI-H358 cells there were more proteins with decreased reversible oxidation as compared with the respective control at 24 h treatment. Interestingly, in the NCI-H358 cells, the top pathways showing statistically significant changes in protein oxidation were associated with the metabolism of fatty acids. Lipidomics studies will need to be performed in the future to inform on the functional consequence of protein oxidation on fatty acids biosynthesis and oxidation.

IPA pointed to the protein sumoylation as one of the top redox pathways regulated by AgNPs in both cell lines. It has been previously shown that sumoylation can be regulated by a redox switch, where a reversible disulfide bond is formed between SUMO-activating enzyme subunit 1 (SAE1/SAE2) and E2-conjugating enzyme UBC9 (48). In our redox proteomic data the SAE1/SAE2 was found to be significantly more oxidized as compared with control cells after 24-h exposure in both cell lines, whereas UBC9 did not show significant changes. The oxidative state of the tRNA charging pathway was also affected in the Calu-1 cells, where many of the aminoacyl-tRNA synthetases were found significantly more oxidized after 24 h exposure. Similarly, the NCI-H358 cells appeared to show slightly more oxidation in aminoacyl-tRNA synthetases at 4 h but these increases did not reach statistical significance. Aminoacyl-tRNA synthetases are essential enzymes that ligate amino acids onto tRNA molecules in a critical step of protein translation (49) and the oxidation of aminoacyl-tRNA synthetases has been shown to inhibit the protein translation accuracy in aminoacyl-tRNA-dependent manner (50, 51, 52, 53).

Earlier studies have shown also that mitochondria are damaged by exposure to AgNPs (5), and when we limited the pathway analysis to only mitochondrial proteins, mitochondrial dysfunction was the top pathway highlighted for both the proteomic and redox proteomic datasets and in both cell lines. Other representative pathways included energy pathways such as oxidative phosphorylation, TCA cycle, fatty acids β-oxidation, and amino acid metabolism. AgNPs affected many of the proteins involved in these pathways either at the protein level or oxidation, which is in line with our earlier results showing that AgNPs exposure reduces ATP-content in both Calu-1 and NCI-H358 cells (5).

Recently, exposure to AgNPs has been shown to change the mitochondrial morphology and ultrastructure as well as the expression of protein involved in mitochondrial fission, fusion, and biogenesis (54, 55). In mitochondrial fission, one mitochondrion is separated in two by the DRP1 and Dnm2 after recruitment of DRP1 to the mitochondria by FIS1, Mff, MiD49, and/or MiD51 (56). Even though FIS1 can act as DRP1 recruiter, it is not required for the recruitment, and overexpression of FIS1 can also induce mitochondrial fission, even in the absence of DRP1 (57). In our proteomics data, we could detect DRP1 and FIS1 fission proteins. DRP1 abundance was higher in the Calu-1 cells than NCI-H358, and it was found increased and highly oxidized after 24 h of AgNPs exposure in Calu-1 cells. In contrast, in the NCI-H358 cells the abundance and oxidation seemed to decrease at 24 h exposure, although these changes were not statistically significant. Posttranslational modifications of DRP1, such as phosphorylation, sumoylation, S-nitrosylation, sulfenylation, or hydropersulfidation (58, 59, 60), have been shown to be important for regulating mitochondrial fission. Interestingly, FIS1 was more abundant at the basal level in the NCI-H358 cells as compared with Calu-1 cells, but AgNPs did not affect the protein level or oxidation. We did not detect any mitochondrial fusion proteins (*e.g.*, Mfn1, Mfn2 or OPA1) in our proteomic or redox proteomic datasets. However, the activity of these proteins is inhibited by FIS1 (61) suggesting increased mitochondrial fusion in Calu-1 and the reverse in NCI-H358, consistent with TEM imaging data. Mfn2 is also known to be targeted for ubiquitin-mediated proteasomal degradation through JNK-phosphorylation in response to cellular stress (62), and as JNK is activated by exposure to AgNPs (63), the results imply further suppression of mitochondrial fusion in AgNPs-treated cells, which is evident in the TEM images of AgNPs-treated NCI-H358 cells. Other mechanisms could involve TRIM28, the phosphorylation of which was shown to downregulate Mfn2 in response to starvation-induced oxidative stress in breast cancer cells (64). We found TRIM28 to be more oxidized in Calu-1 cells at both 4 h and 24 h AgNPs treatment, but the relationship between TRIM28 oxidation and phosphorylation is not currently known and remains to be investigated. Overall, our results on mitochondrial dynamics are well aligned with a recently reported study (55) investigating the effects of AgNPs in rat lungs, whereas they differ from findings in HepG2 liver cancer cells (54), suggesting possible tissue specificities underlying the molecular effects of AgNPs.

We also visualized the changes in the mitochondrial network using confocal imaging and MitoTracker Green staining. The NCI-H358 cells showed less interconnected mitochondria at the basal level with no significant changes noted upon exposure to AgNPs, findings confirmed by TEM imaging. However, the mitochondria in Calu-1 besides occupying a significantly higher footprint were also more elongated at 4 h AgNPs exposure progressing to a more fractured structure after 24 h treatment. TEM analysis provided further insight to these observations showing mitochondrial swelling and loss of integrity with evidence of outer membrane rupture in the AgNPs-treated Calu-1 cells. In addition, striking differences were noted between the Calu-1 and NCI-H358 at baseline with the mitochondria in Calu-1 being more elongated and displaying a less dense stroma and poorly defined cristae in comparison with the mitochondria in NCI-H358 cells. This could be due to NCI-H358 cells exerting better control over basal and AgNPs-induced ROS by controlling ROS production through upregulation of mitochondrial fatty acids β-oxidation over glycolysis (65), and by activating ROS suppression through NRF2 signaling.

In conclusion, AgNPs regulate protein abundance and oxidation in cell-specific fashion affecting many important cellular processes across subcellular organelles, ultimately converging on protein homeostasis and energy metabolism. Furthermore, our work shows that distinct biological responses to AgNPs can be detected at the proteomic level as soon as 4 h after exposure to AgNPs in cells that are sensitive (Calu-1) or insensitive (NCI-H358) to AgNPs-induced cytotoxicity. The precise mechanisms of interorganelle communication to support the coordinated response to AgNPs remain to be investigated but are currently limited by lack of knowledge of functional effects of oxidation on the activity of most proteins identified by our redox proteomic analysis. This gap needs to be addressed to better understand the network-wide effects of redox regulation in determining the sensitivity to environmental or therapeutic AgNPs.

## Data availability

The mass spectrometry proteomics data have been deposited to the ProteomeXchange Consortium *via* the PRIDE (66) partner repository with the data set identifier PXD021493 and 10.6019/PXD021493. Confocal microscopy and TEM data are available on the BioStudies database with the accession S-BSST636 (https://www.ebi.ac.uk/biostudies/studies/S-BSST636?query=S-BSST636).

## Supplemental data

This article contains supplemental data.

## Conflict of interest

C. M. F. is cofounder of Xoder Technologies, LLC, which provides consulting services and commercializes reagents for redox investigations.

## Supplemental Data

Supplemental Data S1A

Supplemental Data S1B
