# Supplementary material for: Integrated Redox Proteomic Analysis Highlights New Mechanisms of Sensitivity to Silver Nanoparticles
Source: Mol Cell Proteomics. 2021 Mar 20;20:100073. doi: 10.1016/j.mcpro.2021.100073 (PMC8724861; doi:10.1016/j.mcpro.2021.100073)
Supplement: Supplemental Figures S1–S4 [file mmc7.docx]

# Supplementary Material

# Integrated Redox Proteomic Analysis Highlights New Mechanisms of Sensitivity to Silver Nanoparticles

Reetta Holmila^1^, Hanzhi Wu^1,2^, Jingyun Lee^2^, Allen W. Tsang^1,2,3^, Ravi Singh^2,3,4^, Cristina M. Furdui^1,2,3,*^

^1^ Department of Internal Medicine, Section on Molecular Medicine, Wake Forest School of Medicine, Winston-Salem, North Carolina 27157

^2^ Wake Forest Baptist Comprehensive Cancer Center, Wake Forest Baptist Medical Center, Winston-Salem, North Carolina, 27157

^3^ Center for Redox Biology and Medicine, Wake Forest School of Medicine, Winston-Salem, North Carolina, 27157

^4^ Department of Cancer Biology, Wake Forest School of Medicine, Winston-Salem, North Carolina 27157,

* Corresponding Author: [cfurdui@wakehealth.edu](mailto:cfurdui@wakehealth.edu)

**Running Title:** Redox and Proteomics Effects of AgNPs

**Keywords:** Silver Nanoparticles, Lung, Proteomics, Reversible Protein Oxidation, Mitochondria

**Supplementary Figures**

**
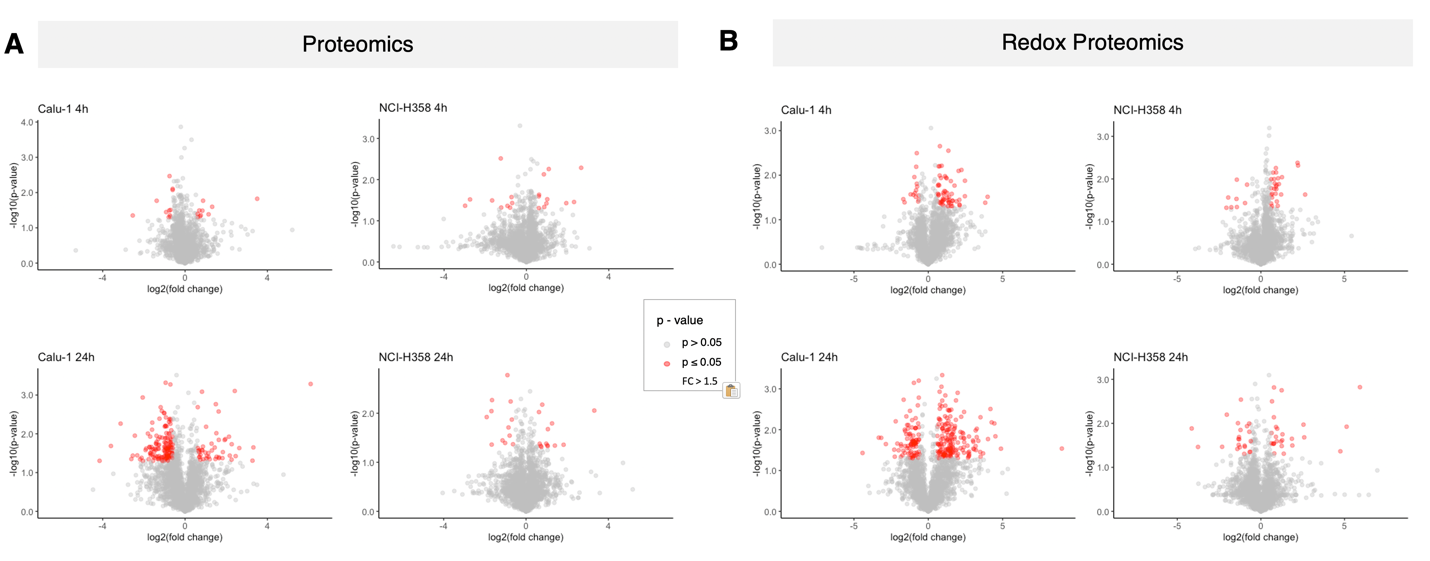
**

**Supplementary Figure S1. Comparison analysis of significantly altered proteins (p<0.05; FC>1.5) induced by treatment with AgNPs.**

A Volcano plots showing proteins with significantly altered abundance at 4h and 24h treatment with AgNPs.

B Volcano plots showing proteins with significantly altered redox state at 4h and 24h treatment with AgNPs.

Data Information: Red symbols represent statistically significant changes (Student’s t-test, p<0.05 and fold change FC>1.5, n = 3) in protein abundance (A), redox state (B), in the two cell lines at the two treatment time points relative to their respective controls.


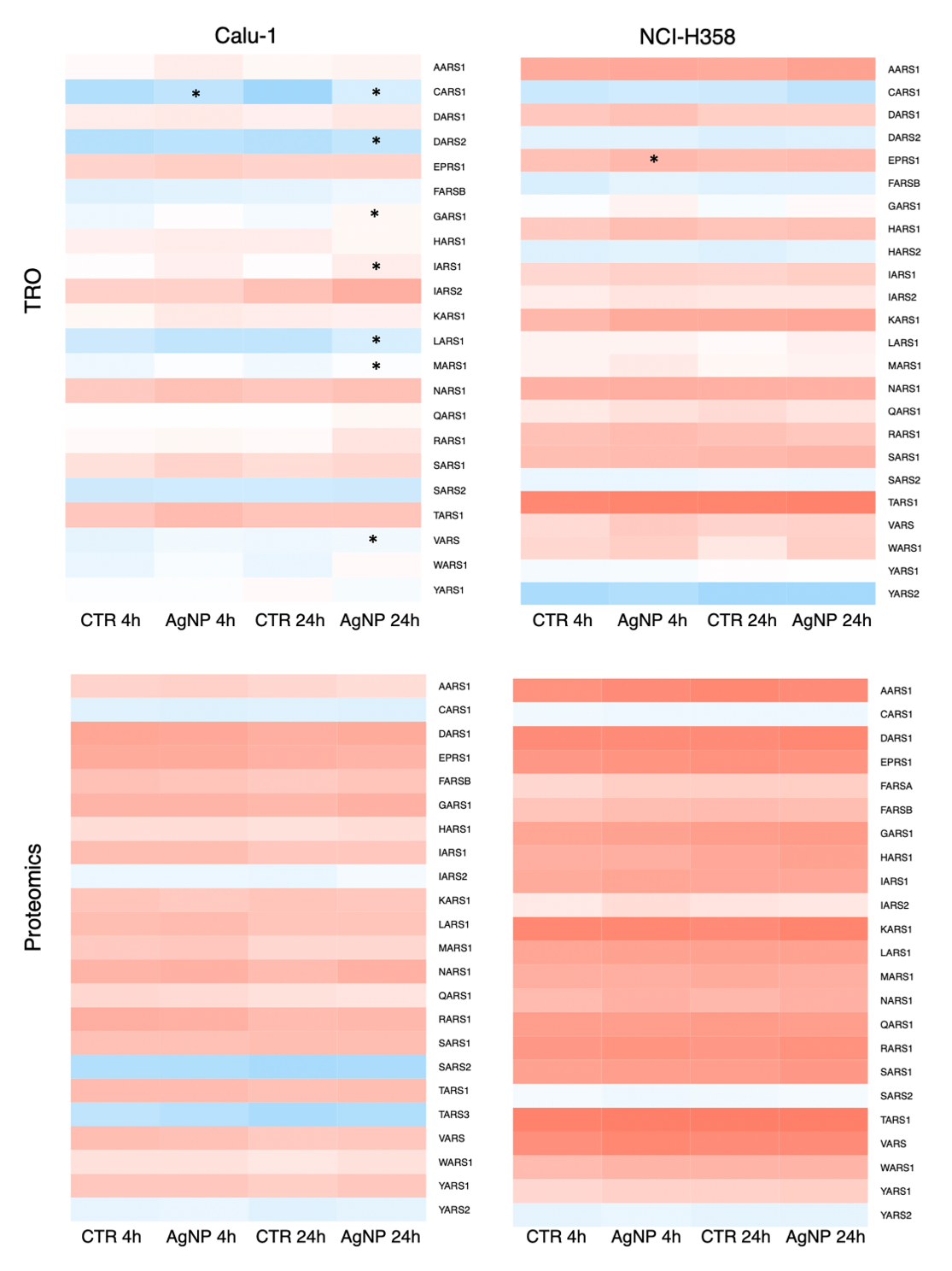


**Supplementary Figure S2 -** **Heatmap of proteins involved in aminoacyl-tRNA synthesis.**

A Heatmap of redox proteomics (TRO) data. * shows the statistically significant changes (p < 0.05), calculated using Student’s paired t-test and comparing AgNPs exposed cells to the respective control conditions.

B Heatmap of proteomics data showing the changes in redox state are not due to changes in protein expression.

**
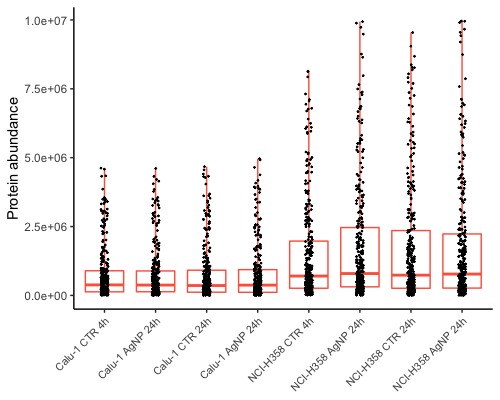
**

**Supplementary Figure S3. Distribution of mitochondrial proteins abundance.** Mitochondrial proteins are defined as proteins listed as mitochondrial in the MitoCarta database.


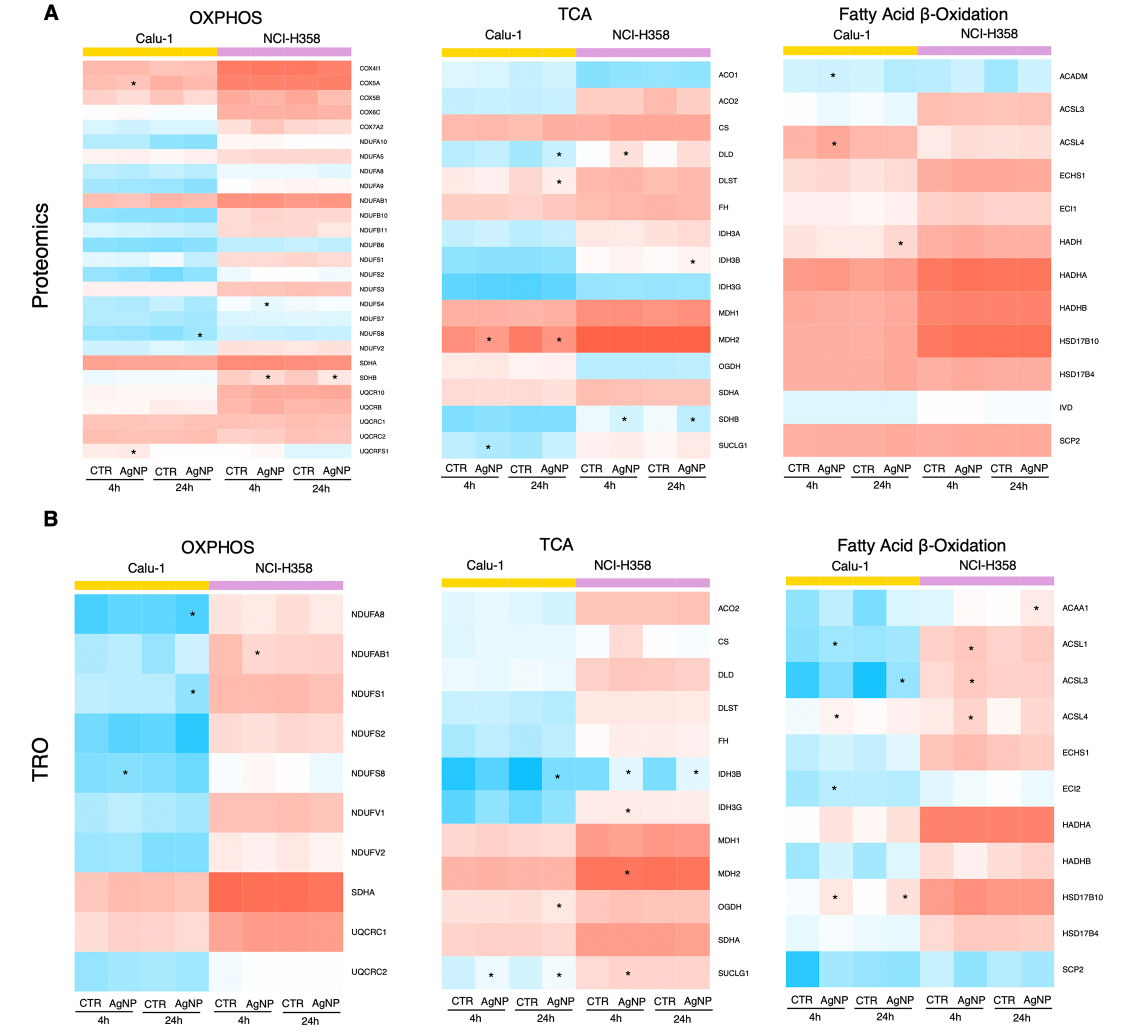


**Supplementary Figure S4 - Heatmaps for proteins involved in energy pathways.**

A Heatmap analysis using proteomic data.

B Heatmap analysis using redox proteomic data (TRO).

In both panels A and B, * shows the statistically significant changes (p < 0.05) between AgNPs exposed cells and the respective controls, calculated using Student’s paired t-test.
